# Divergent effects of oral health and genetic risk: competing risks of dementia and mortality

**DOI:** 10.1093/geroni/igag052

**Published:** 2026-05-07

**Authors:** Ruotong Liu, Xiang Qi, Yunrui Liu, Huabin Luo, Zhijing Xu, Bei Wu

**Affiliations:** Rory Meyers College of Nursing, New York University, New York, New York, United States; Rory Meyers College of Nursing, New York University, New York, New York, United States; Rory Meyers College of Nursing, New York University, New York, New York, United States; Department of Public Health, East Carolina University, Greenville, North Carolina, United States; Rory Meyers College of Nursing, New York University, New York, New York, United States; Division of Arts and Sciences, NYU Shanghai, Shanghai, China

**Keywords:** Edentulism, Polygenic risk score, Gene–environment interaction

## Abstract

**Background and Objectives:**

Polygenic risk scores (PRS) for dementia are increasingly used to capture genetic susceptibility, yet their predictive utility may depend on coexisting health and social conditions. Edentulism, an extreme marker of oral health deterioration and accumulated disadvantage, may influence how genetic risk manifests in later life. This study aims to investigate the independent and joint associations of edentulism and dementia PRS with incident all-cause dementia and mortality risk.

**Research Design and Methods:**

We analyzed longitudinal data from the Health and Retirement Study linked to Medicare claims. The sample included 9,806 dementia-free participants aged ≥67 with information on edentulism and PRS for Alzheimer’s disease. Edentulism was self-reported and logically imputed across waves. PRS was categorized as low, intermediate, and high. Outcomes included incident dementia and all-cause mortality. Cox proportional hazards and Fine–Gray competing risk models were used to examine independent and interactive associations.

**Results:**

Edentulism was not independently associated with dementia risk after full adjustment. High PRS was associated with increased dementia risk (subdistribution hazard ratio [sHR] 1.20; 95% confidence interval [CI], 1.06-1.39). However, the association between PRS and dementia was weaker among edentulous participants (interaction sHR 0.73; 95% CI, 0.54-0.99). In contrast, mortality risk was higher among individuals with both edentulism and high PRS (interaction sHR 1.36; 95% CI, 1.00-1.84).

**Discussion and Implications:**

These findings suggest that oral health status may modify the association between genetic risk and dementia in late life. Mortality may occur before a diagnosis of dementia, underscoring the importance of considering competing health risks when interpreting genetic susceptibility in aging populations.

Innovation and Translational Significance: This study integrates oral health and genetic susceptibility within a competing risk framework to examine how systemic health conditions influence dementia risk manifestation. Using nationally representative longitudinal data linked to genetic and Medicare claims information, we show that the association between polygenic risk and incident dementia differs by oral health status and is shaped by competing mortality risk. It highlights the importance of considering mortality as a competing risk of dementia when interpreting genetic risk in aging populations. Results underscore the need for future research integrating genetic information with life-course health conditions to better understand heterogeneity in dementia risk.

## Background

Dementia represents one of the most pressing public health challenges of aging societies, with profound implications for longevity, functional independence, and healthcare burden.^1–3^ Although genetic susceptibility, particularly as quantified by polygenic risk scores (PRS), plays a key role in dementia pathogenesis, it remains insufficient to explain observed variation in dementia risk across populations and contexts.^4^ Increasing evidence points to modifiable factors, including vascular, metabolic, and oral health conditions, that may interact with genetic vulnerability to shape cognitive aging.^5–7^

The translation of dementia-related PRS from research tools into practical applications in population health and aging research represents a key next step in advancing our understanding of genetic susceptibility. However, a critical barrier to this translation is the incomplete understanding of how genetic risk is expressed within heterogeneous populations, each shaped by distinct health, behavioral, and socioeconomic profiles.^6^ A high PRS does not confer a uniform risk; its clinical manifestation depends on an individual’s accumulated life-course exposures, physiological resilience, and competing health burdens.^8^^,^^9^ Identifying factors that either amplify or attenuate the impact of genetic predisposition is thus essential for both risk stratification and targeted intervention.

Oral health, edentulism in particular, has emerged as a promising yet understudied factor in this regard. Edentulism, the complete loss of all natural teeth, embodies cumulative disadvantage, chronic inflammation, nutritional compromise, and limited access to preventive care, which reflects a lifetime exposure to systemic and structural vulnerabilities.^10^^,^^11^ Epidemiologic evidence has linked tooth loss to cognitive decline and dementia, potentially through inflammatory, vascular, and metabolic pathways.^12–18^ Yet, how tooth loss affects dementia for persons with distinct genetic or clinical profiles remains underexplored.

Edentulism offers a unique lens for studying gene–environment interplay. It represents a concrete and clinically meaningful indicator of systemic frailty and physiological wear.^19^^,^^20^ Clarifying its interaction with genetic risk could reveal whether the impact of a high dementia PRS is most pronounced among otherwise healthy individuals or is amplified among those with substantial health burdens and reduced biological reserve.

Furthermore, any investigation of dementia risk in older adults must contend with the competing risk of mortality.^21^^,^^22^ Individuals with the greatest health burdens, such as those who are edentulous, face an elevated likelihood of death from multiple causes before dementia diagnosis can occur.^23^ Analyses that fail to account for this competing risk may misrepresent the true direction and magnitude of associations. Accordingly, treating mortality as a distinct yet interrelated outcome is essential to accurately interpret associations between health exposures and dementia.

In this study, we aim to investigate the independent and joint associations of edentulism and dementia PRS with two competing outcomes: incident all-cause dementia and mortality risk. We hypothesized that both edentulism and higher PRS would be independently associated with increased risks of dementia and mortality, but their combined effects would diverge: attenuating dementia risk through competing mortality while amplifying mortality risk through synergistic health burdens.

## Method

### Study design, setting, and participants

This cohort study drew data from the Health and Retirement Study (HRS), a nationally representative longitudinal panel study of U.S. adults aged 50 and older. The HRS conducts biennial interviews to collect comprehensive data on health, cognition, and socioeconomic factors.^24^ For consenting participants (approximately 88%), these survey data were linked to Medicare claims records from the Center for Medicare & Medicaid Services, providing detailed diagnostic information from inpatient, outpatient, and other healthcare settings.^25^

Genetic data were available for a subset of participants who provided a salivary sample for genotyping between 2006 and 2012. From these data, the HRS team constructed and provided a series of PRS for analysis. Information on edentulism was first collected in 2006 and subsequently every 6 years. To establish incident (first onset) dementia, a 2-year “washout” period was applied, examining Medicare claims from 2004 onward to confirm no prior dementia diagnoses before the study baseline. The observation period extended through 2021 to ascertain all dementia diagnoses and deaths.

**Figure 1:**
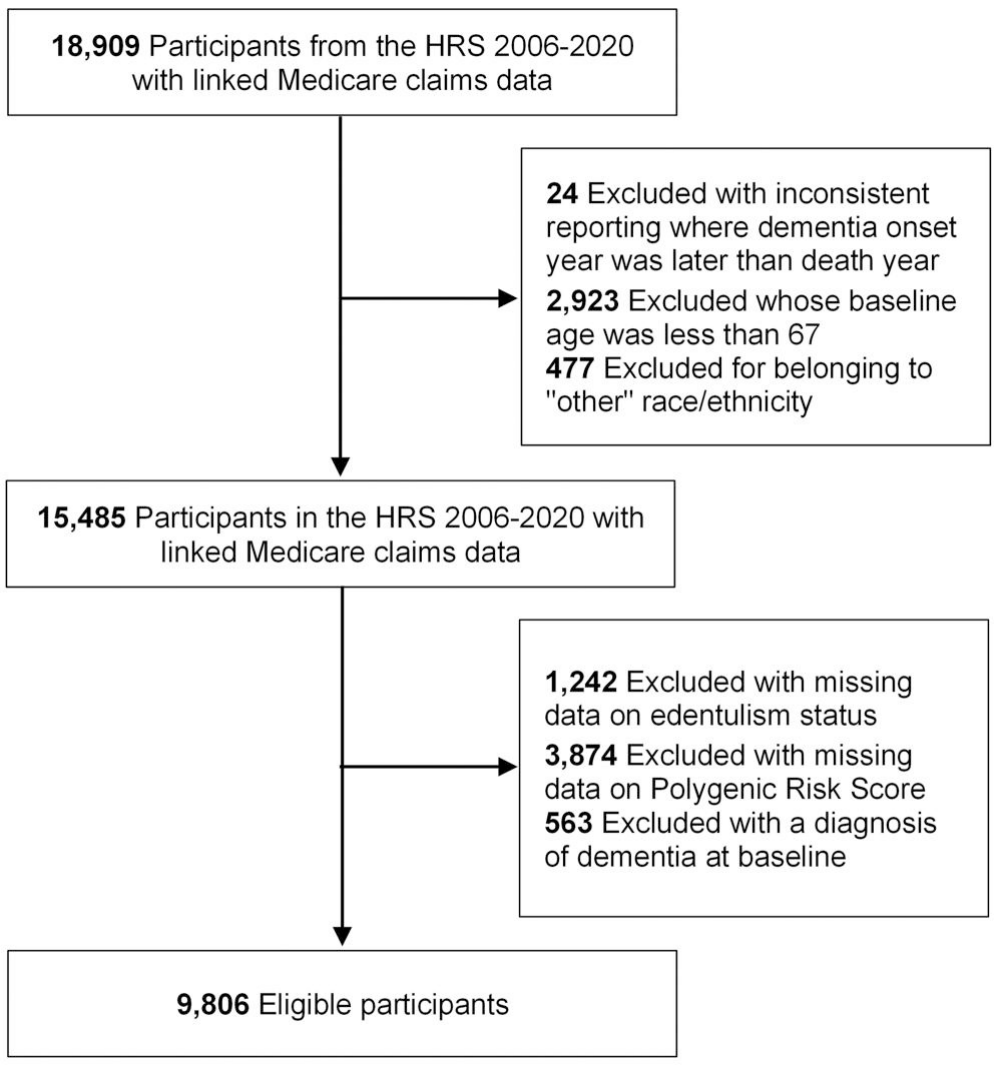
Flowchart of study sample selection. HRS, Health and Retirement Study.

To maximize sample inclusion, an individual-level baseline was established. For each participant, the baseline was defined as their first survey wave occurring at or after the age of 67. The initial sample comprised 18,909 participants. As detailed in the sample selection flowchart (Figure 1), we sequentially excluded participants with inconsistent reporting of dementia and death dates (*n* = 24), those younger than 67 at their first eligible wave (*n* = 2923), individuals who identified as a race/ethnicity other than non-Hispanic White, non-Hispanic Black, or Hispanic due to unavailable PRS data (*n* = 477), and those with missing data on edentulism (*n* = 1242). Of the remaining cohort, 10,369 had available PRS data. Finally, to restrict the analysis to incident cases, we excluded participants with prevalent dementia at baseline (*n* = 563). This process yielded a final analytical cohort of 9806 dementia-free participants.

### Outcomes

#### Incident dementia

Incident all-cause dementia was ascertained using a validated algorithm applied to linked Medicare claims data (inpatient, outpatient, skilled nursing facility, home health, and carrier files). This algorithm enhances specificity by requiring a combination of diagnosis and symptom codes based on the International Classification of Diseases, 9th/10th Revision (ICD-9/ICD-10; Table S1). The algorithm’s criteria included a 2-year washout period to establish incidence and a subsequent confirmatory code or death to validate the diagnosis.^26^^,^^27^ The date of the first qualifying dementia code was defined as the date of dementia onset.

#### All-cause mortality

Mortality data, including the year of death, were ascertained from Medicare records.

### Exposure variables

#### Edentulism

Edentulism was operationalized as the self-reported complete loss of all natural teeth. This was assessed in the 2006, 2012, and 2018 HRS Core questionnaire via the question, “Have you lost all of your upper and lower permanent teeth?” For interim waves (2008, 2010, 2014, 2016, and 2020), dentition status was imputed based on the logical assumption that edentulism is an irreversible state.^10^^,^^28^ Specifically, if a participant reported being edentulous at any time point, this status was carried forward to all subsequent waves; similarly, dentate status was carried backward when consistently reported.

#### Polygenic risk score

The PRS for Alzheimer’s disease (AD) was obtained directly from pre-computed, quality-controlled data provided by the HRS. This score was derived from the 2019 International Genomics of Alzheimer’s Project (IGAP) genome-wide association study (GWAS) discovery sample.^29^ To maximize predictive power, the AD PRS was constructed using single-nucleotide polymorphisms with a *p* value <.01 in the source GWAS. The HRS calculated this score separately for participants who self-identified as non-Hispanic White, non-Hispanic Black, or Hispanic. Consistent with prior research,^30^ we categorized the continuous PRS into quintiles and then collapsed these into three risk groups for analysis: low (the lowest quintile), intermediate (quintiles 2-4), and high (the highest quintile).

### Covariates

Based on established literature,^31^^,^^32^ we included a comprehensive set of covariates measured at baseline. Sociodemographic variables included age (years), sex (male, female), race/ethnicity (non-Hispanic White, non-Hispanic Black, Hispanic), education (years), marital status (married/partnered vs other), household wealth (wave-specific tertiles), and Medicaid enrollment status. Health behaviors included self-reported dental visits (within the past 2 years vs not), physical exercise (≥once/month), cigarette smoking (ever vs never), alcohol consumption (ever vs never), and body mass index (BMI), categorized as underweight (BMI < 18.5), normal weight (18.5≤BMI < 25), overweight (25≤BMI < 30), and obese (BMI ≥ 30). Health conditions included self-reported physician-diagnosed heart disease, stroke, and diabetes.

### Statistical analysis

First, we summarized the baseline characteristics of the analytical sample, stratified by edentulism status. Group differences were evaluated using an independent *t*-test for continuous variables and χ^2^ tests for categorical variables. To assess potential selection bias, we then used these same tests to compare the baseline characteristics of the analytical cohort (participants dementia-free at baseline) with those who were excluded due to a prevalent dementia diagnosis.

The primary analyses used survival modeling to separately examine the associations between the exposures and two key outcomes: incident all-cause dementia and mortality risk. Recognizing that death can act as a competing event for dementia because it precludes the subsequent observation of dementia, we employed two complementary modeling approaches to appropriately address this issue. First, we used Cox proportional hazards models to estimate cause-specific hazard ratios (HRs), which represent the instantaneous rate of a specific event (eg, dementia) among individuals who have not yet experienced either event. Second, to directly model the cumulative incidence, we utilized Fine–Gray competing risk models. This approach yields the subdistribution hazard ratios (sHRs), which quantify the association between an exposure and the overall probability that an event will occur over time, while accounting for the fact that some individuals will experience the competing event instead. When incident dementia was the outcome, death was treated as the competing risk. For all models, analysis time began at the baseline interview and ended at the date of first dementia diagnosis, death, loss to follow-up, or the end of the study period in 2021, whichever occurred first.

We constructed a series of nested regression models to systematically examine the independent and interactive effects of our primary exposures. Our initial model (Model 1) included edentulism status as the main exposure to evaluate its association with the outcomes. Next, we added the PRS to assess its independent association while controlling for edentulism in Model 2. Finally, to test for effect modification, we introduced a multiplicative interaction term between edentulism status and PRS in Model 3. All models were fully adjusted for the complete set of sociodemographic, health behavior, and health condition covariates.

As a sensitivity analysis (Model 4), we replicated our final interaction model using age as the underlying time metric instead of time since baseline. All models handled missing covariate data via listwise deletion, as the proportion of missing data on covariates was minimal (*n* = 160; 1.63%). A two-sided *p* value of <.05 was considered statistically significant. All statistical procedures were performed using Stata/MP 19.0 (StataCorp LLC, College Station, TX) within a secure data Enclave.

## Results

### Baseline sample characteristics

Of the 9806 participants who were dementia-free at baseline, 1901 (19.4%) were edentulous, 2427 (24.8%) developed incident dementia during follow-up, and 2429 (24.8%) died without a recorded dementia diagnosis. As shown in Table 1, the mean baseline age of participants was 72.2 (*SD* = 6.1) years, and 57.8% were female. The sample was predominantly non-Hispanic White (77.3%), with 13.6% non-Hispanic Black and 9.0% Hispanic participants. Compared to their dentate counterparts, edentulous individuals were older, less educated, had lower household wealth, and were more likely to be non-Hispanic Black, unmarried, and enrolled in Medicaid (*p* < .001 for all). Edentulous participants also reported lower rates of recent dental visits and physical exercise, higher rates of ever-smoking, heart disease, stroke, and diabetes (*p* < .001 for all). The distribution of PRS categories did not differ significantly between the two groups (*p* = .846).

**Table 1:** Baseline characteristics of the analytical sample by edentulism status.

Variables	All	Dentate	Edentulous	*p*
	*N *= 9806	*n *= 7905 (80.61%)	*n *= 1901 (19.39%)	
**Polygenic risk score**				.846
Low	1972 (20.11%)	1596 (20.19%)	376 (19.78%)	
Intermediate	5909 (60.26%)	4765 (60.28%)	1144 (60.18%)	
High	1925 (19.63%)	1544 (19.53%)	381 (20.04%)	
**Age (67-101)**	72.21 (6.11)	72.03 (5.98)	72.99 (6.55)	<.001
**Female**	5666 (57.78%)	4539 (57.42%)	1127 (59.28%)	.139
**Race**				<.001
Non-Hispanic White	7584 (77.34%)	6235 (78.87%)	1349 (70.96%)	
Non-Hispanic Black	1337 (13.63%)	967 (12.23%)	370 (19.46%)	
Hispanic	885 (9.03%)	703 (8.89%)	182 (9.57%)	
**Education (0-17)**	12.47 (3.17)	12.84 (3.08)	10.91 (3.05)	<.001
**Married**	6373 (65.00%)	1054 (55.44%)	5319 (67.30%)	<.001
**Household wealth**				<.001
1st	2707 (27.61%)	1814 (22.95%)	893 (46.98%)	
2nd	3304 (33.69%)	2626 (33.22%)	678 (35.67%)	
3rd	3795 (38.70%)	3465 (43.83%)	330 (17.36%)	
**Medicaid**	774 (7.94%)	493 (6.26%)	281 (14.98%)	<.001
**Dental visit**	6202 (63.36%)	5856 (74.16%)	346 (18.29%)	<.001
**Exercise ≥once/month**	7783 (79.48%)	6478 (82.07%)	1305 (68.68%)	<.001
**Smoking (ever)**	5551 (57.02%)	4212 (53.68%)	1339 (70.85%)	<.001
**Drinking (ever)**	5078 (51.80%)	4372 (55.33%)	706 (37.14%)	<.001
**Body mass index**				.001
Normal weight	2745 (27.99%)	2233 (28.25%)	512 (26.93%)	
Underweight	117 (1.19%)	82 (1.04%)	35 (1.84%)	
Overweight	3771 (38.46%)	3083 (39.00%)	688 (36.19%)	
Obese	3173 (32.36%)	2507 (3171%)	666 (35.03%)	
**Heart disease**	2614 (26.66%)	1989 (25.16%)	625 (32.88%)	<.001
**Stroke**	790 (8.06%)	570 (7.21%)	220 (11.57%)	<.001
**Diabetes**	2217 (22.61%)	1683 (21.29%)	534 (28.09%)	<.001
**Outcome**				<.001
Incident dementia	2427 (24.75%)	1895 (23.97%)	532 (27.99%)	
Death	2429 (24.77%)	1766 (22.34%)	663 (34.88%)	
Censorship	4950 (50.48%)	4244 (53.69%)	706 (37.14%)	

Data are expressed as Mean (Standard Deviation [SD]) for continuous variables and *N* (%) for categorical variables. Percentages have been rounded and may not total 100, and numbers may not total numbers in column headings owing to missing data. Comparisons were performed using a t-test for continuous variables and the χ^2^ tests for categorical variables.

To assess for potential selection bias, we compared the analytical cohort with participants who were excluded due to prevalent dementia at baseline (*n* = 563; Table S2). The excluded group was significantly older, had fewer years of education, and had a higher prevalence of edentulism. They also exhibited a greater burden of heart disease and stroke. The distribution of sex, race/ethnicity, and PRS did not significantly differ between the included and excluded groups.

### Association of edentulism and PRS with incident dementia

Results from the Cox and Fine–Gray models for incident dementia are presented in Table 2. In the initial Cox model adjusted for covariates (Model 1), edentulism was not significantly associated with the risk of dementia (HR = 1.04; 95% confidence interval [CI], 0.94-1.15). After additionally controlling for PRS (Model 2), the association for edentulism remained unchanged. In the final model with the interaction of edentulism * PRS (Model 3), a significant negative interaction between edentulism and high PRS was revealed (interaction HR = 0.73; 95% CI, 0.55-0.96), indicating that the association between high PRS and dementia was weaker among individuals with edentulism, a pattern that may reflect survival dynamics under competing mortality.

**Table 2:** Associations of edentulism and PRS with incident all-cause dementia.

Variables	Cox proportional hazards models				Fine–Gray competing risk models^a^			
	Hazard ratios (95% CI)				Subdistribution hazard ratios (95% CI)			
	Model 1	Model 2	Model 3	Model 4	Model 1	Model 2	Model 3	Model 4
**Edentulism**	1.04 (0.94, 1.15)	1.04 (0.94, 1.15)	1.23 (1.00, 1.50)	1.23 (1.00, 1.51)	1.01 (0.90, 1.13)	1.01 (0.90, 1.13)	1.18 (0.95, 1.47)	1.23 (0.99, 1.53)
**PRS (ref: low)**								
Intermediate	—	1.00 (0.90, 1.10)	1.04 (0.93, 1.16)	1.03 (0.92, 1.16)	—	0.99 (0.90, 1.09)	1.03 (0.92, 1.15)	0.97 (0.87, 1.09)
High		1.12 (0.99, 1.26)	1.20 (1.05, 1.37)	1.20 (1.05, 1.37)		1.13 (1.00, 1.27)	1.21 (1.06, 1.39)	1.16 (1.02, 1.33)
Edentulism * PRS								
Intermediate	—	—	0.85 (0.67, 1.07)	0.85 (0.67, 1.07)	—	—	0.85 (0.67, 1.09)	0.82 (0.64, 1.05)
High			0.73 (0.55, 0.96)	0.74 (0.56, 0.98)			0.73 (0.54, 0.99)	0.70 (0.52, 0.94)
**Age**	1.11 (1.10, 1.11)	1.11 (1.10, 1.11)	1.11 (1.10, 1.11)	—	1.08 (1.08, 1.09)	1.08 (1.08, 1.09)	1.08 (1.08, 1.09)	—
**Female**	0.97 (0.89, 1.06)	0.97 (0.89, 1.07)	0.97 (0.89, 1.06)	0.98 (0.90, 1.07)	1.09 (0.99, 1.19)	1.09 (1.00, 1.19)	1.09 (1.00, 1.19)	1.04 (0.95, 1.14)
**Race (ref: Non-Hispanic White)**								
Non-Hispanic Black	1.05 (0.92, 1.19)	1.05 (0.92, 1.19)	1.05 (0.93, 1.19)	1.07 (0.94, 1.21)	1.08 (0.95, 1.23)	1.08 (0.95, 1.23)	1.08 (0.95, 1.23)	1.03 (0.90, 1.17)
Hispanic	0.77 (0.65, 0.92)	0.77 (0.65, 0.92)	0.77 (0.65, 0.92)	0.79 (0.66, 0.94)	0.85 (0.71, 1.00)	0.85 (0.71, 1.01)	0.85 (0.71, 1.01)	0.79 (0.66, 0.94)
**Education**	0.99 (0.97, 1.00)	0.99 (0.97, 1.00)	0.99 (0.97, 1.00)	0.99 (0.98, 1.01)	0.99 (0.98, 1.01)	0.99 (0.98, 1.01)	0.99 (0.98, 1.01)	0.98 (0.97, 1.00)
**Married**	1.05 (0.96, 1.15)	1.05 (0.96, 1.15)	1.05 (0.96, 1.15)	1.04 (0.95, 1.13)	1.06 (0.97, 1.16)	1.06 (0.97, 1.16)	1.06 (0.97, 1.16)	0.85 (0.78, 0.93)
**Household wealth**								
2^nd^	0.84 (0.75, 0.93)	0.84 (0.75, 0.93)	0.84 (0.75, 0.94)	0.85 (0.76, 0.94)	0.87 (0.78, 0.97)	0.86 (0.77, 0.96)	0.86 (0.77, 0.96)	0.91 (0.81, 1.01)
3^rd^	0.82 (0.73, 0.93)	0.82 (0.73, 0.92)	0.82 (0.73, 0.93)	0.83 (0.73, 0.93)	0.85 (0.75, 0.96)	0.85 (0.75, 0.95)	0.85 (0.75, 0.96)	0.91 (0.80, 1.02)
**Medicaid**	1.09 (0.93, 1.28)	1.08 (0.93, 1.27)	1.09 (0.93, 1.27)	1.10 (0.94, 1.28)	1.07 (0.90, 1.26)	1.06 (0.90, 1.26)	1.06 (0.90, 1.26)	0.99 (0.84, 1.17)
**Dental visit**	0.96 (0.87, 1.05)	0.96 (0.87, 1.05)	0.96 (0.87, 1.05)	0.96 (0.88, 1.06)	1.02 (0.93, 1.13)	1.02 (0.93, 1.13)	1.02 (0.93, 1.13)	1.05 (0.95, 1.16)
**Exercise ≥once/month**	0.68 (0.62, 0.74)	0.68 (0.62, 0.75)	0.68 (0.62, 0.75)	0.69 (0.63, 0.75)	0.81 (0.74, 0.90)	0.81 (0.74, 0.90)	0.81 (0.74, 0.90)	0.71 (0.65, 0.78)
**Smoking (ever)**	1.20 (1.10, 1.30)	1.20 (1.10, 1.30)	1.19 (1.10, 1.30)	1.19 (1.09, 1.29)	1.09 (1.00, 1.18)	1.09 (1.00, 1.18)	1.08 (1.00, 1.18)	0.95 (0.88, 1.04)
**Drinking (ever)**	0.85 (0.78, 0.93)	0.85 (0.78, 0.92)	0.85 (0.78, 0.92)	0.85 (0.78, 0.93)	0.87 (0.80, 0.94)	0.87 (0.80, 0.94)	0.87 (0.80, 0.94)	0.87 (0.80, 0.94)
**BMI (ref: normal weight)**								
Underweight	1.14 (0.83, 1.57)	1.15 (0.84, 1.58)	1.14 (0.83, 1.57)	1.13 (0.82, 1.55)	0.81 (0.56, 1.18)	0.82 (0.57, 1.20)	0.81 (0.56, 1.19)	0.91 (0.65, 1.28)
Overweight	0.82 (0.75, 0.90)	0.83 (0.75, 0.91)	0.83 (0.75, 0.90)	0.83 (0.76, 0.91)	0.89 (0.81, 0.98)	0.89 (0.81, 0.98)	0.89 (0.81, 0.98)	0.82 (0.75, 0.90)
Obese	0.75 (0.67, 0.83)	0.75 (0.68, 0.83)	0.75 (0.67, 0.83)	0.76 (0.68, 0.84)	0.79 (0.71, 0.88)	0.79 (0.71, 0.88)	0.79 (0.71, 0.88)	0.64 (0.58, 0.72)
**Heart disease**	1.22 (1.12, 1.33)	1.22 (1.12, 1.33)	1.22 (1.12, 1.33)	1.21 (1.11, 1.31)	1.08 (0.99, 1.18)	1.08 (0.99, 1.18)	1.08 (0.99, 1.18)	1.15 (1.05, 1.25)
**Stroke**	1.61 (1.43, 1.80)	1.60 (1.43, 1.80)	1.61 (1.43, 1.81)	1.57 (1.40, 1.77)	1.42 (1.25, 1.61)	1.42 (1.25, 1.61)	1.43 (1.26, 1.62)	1.51 (1.33, 1.71)
**Diabetes**	1.29 (1.17, 1.42)	1.29 (1.17, 1.42)	1.29 (1.17, 1.42)	1.28 (1.17, 1.41)	1.14 (1.03, 1.27)	1.14 (1.03, 1.26)	1.14 (1.03, 1.26)	1.06 (0.96, 1.17)

Abbreviations: BMI, body mass index; PRS, polygenic risk score.

a Competing risk: All-cause mortality.

**Figure 2:**
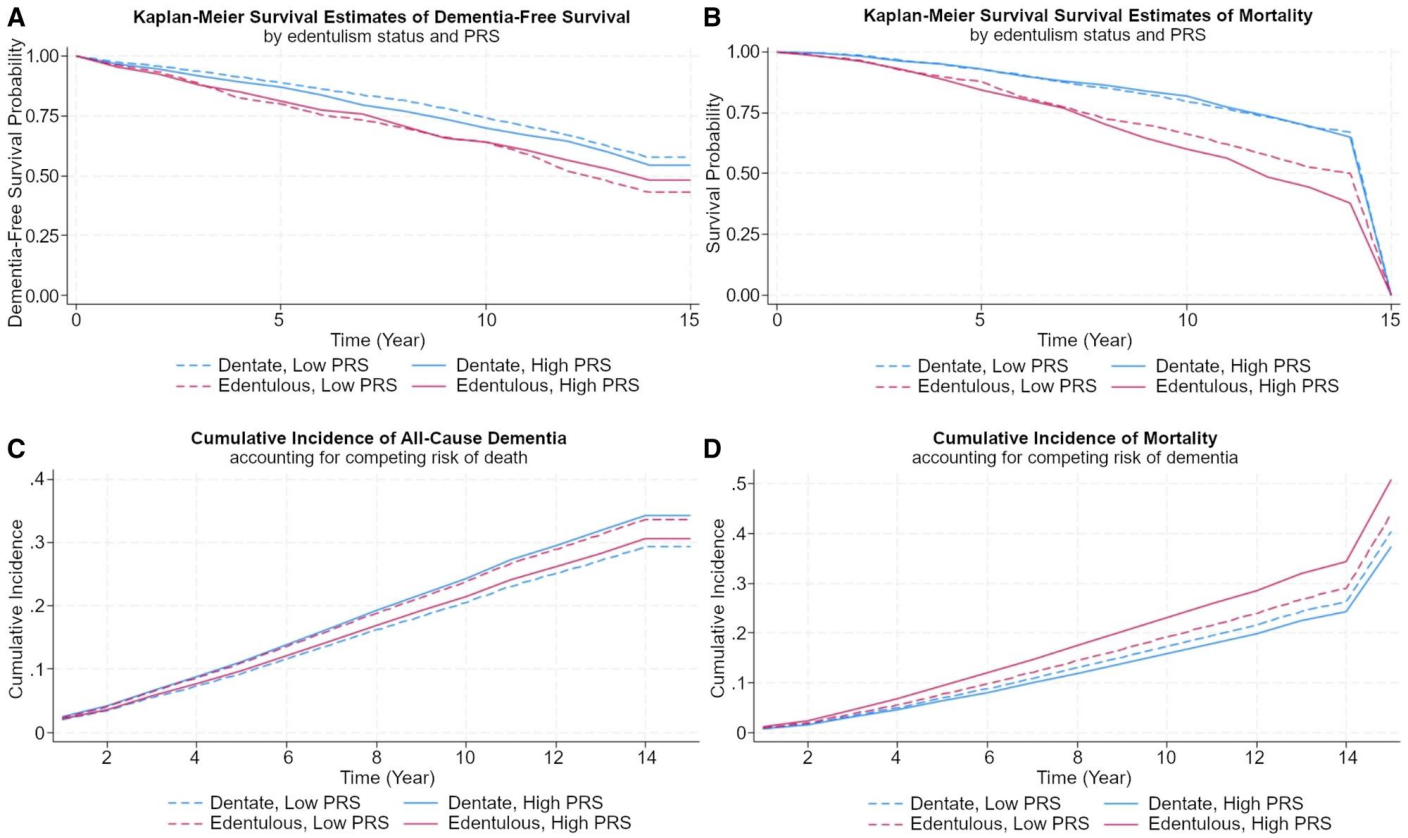
Kaplan–Meier and Fine–Gray estimates of dementia and mortality by edentulism and PRS. PRS, polygenic risk score. Panels A and B display unadjusted Kaplan–Meier survival curves showing dementia-free survival and overall survival probabilities over a 15-year follow-up period. These curves are stratified by edentulism states (dentate vs edentulous) and PRS category (low vs high). Panels C and D display cumulative incidence functions (CIFs) derived from fully adjusted Fine–Gray competing risk models (Model 3 in Tables 2 and 3). Panel C presents the cumulative incidence of all-cause dementia, treating death as a competing risk. Panel D presents the cumulative incidence of all-cause mortality, treating dementia as a competing event. Curves are stratified by four exposure groups representing the combination of edentulism status (dentate vs edentulous) and PRS category (low vs high).

The Fine–Gray competing risk models, which accounted for mortality as a competing event, produced highly consistent findings. In the final interaction model (Model 3), a high PRS was associated with a 21% increase in the risk of dementia (subdistribution hazard ratio [sHR] = 1.21; 95% CI, 1.06-1.39). Corroborating the Cox model, a significant interaction was observed between edentulism and high PRS (sHR = 0.73; 95% CI, 0.54-0.99), demonstrating that the effect of high genetic risk on dementia was diminished in the presence of edentulism. These results are visualized in Figure 2. Panel A presents Kaplan–Meier estimates of dementia-free survival, while Panel C shows cumulative incidence functions from the Fine–Gray competing risk model accounting for death. The curves illustrate the four joint exposure groups defined by dentate status and PRS category, showing that the association between high PRS and increased dementia incidence is most apparent among dentate individuals, whereas the difference between PRS groups is attenuated among those who are edentulous.

### Association of edentulism and PRS with all-cause mortality

For the outcome of all-cause mortality, Cox models showed that edentulism was a significant predictor of mortality in models adjusted for covariates (Model 1; HR = 1.33; 95% CI, 1.19-1.48) and further adjusted for PRS (Model 2; HR = 1.33; 95% CI, 1.19-1.48) (Table 3). In these models, PRS was not significantly associated with mortality. When interaction terms were added (Model 3), no significant interactions were detected, and the main effect of edentulism was attenuated and no longer statistically significant.

**Table 3:** Association of edentulism and PRS with all-cause mortality.

Variables	Cox proportional hazards models				Fine–Gray competing risk models^a^			
	Hazard ratios (95% CI)				Subdistribution hazard ratios (95% CI)			
	Model 1	Model 2	Model 3	Model 4	Model 1	Model 2	Model 3	Model 4
**Edentulism**	1.33 (1.19, 1.48)	1.33 (1.19, 1.48)	1.21 (0.96, 1.53)	1.15 (0.93, 1.43)	1.30 (1.16, 1.45)	1.30 (1.16, 1.45)	1.12 (0.88, 1.42)	1.12 (0.89, 1.42)
**PRS (ref: low)**								
Intermediate	—	1.09 (0.98, 1.22)	1.07 (0.95, 1.21)	1.14 (1.01, 1.28)	—	1.11 (0.99, 1.23)	1.07 (0.94, 1.21)	1.05 (0.92, 1.19)
High		0.99 (0.87, 1.13)	0.93 (0.80, 1.09)	0.99 (0.86, 1.15)		0.99 (0.86, 1.13)	0.91 (0.78, 1.06)	0.88 (0.75, 1.04)
Edentulism * PRS								
Intermediate	—	—	1.08 (0.84, 1.39)	1.06 (0.83, 1.34)	—	—	1.15 (0.89, 1.49)	1.13 (0.87, 1.46)
High			1.25 (0.93, 1.69)	1.17 (0.88, 1.55)			1.36 (1.00, 1.84)	1.40 (1.03, 1.90)
**Age**	1.07 (1.06, 1.08)	1.07 (1.06, 1.08)	1.07 (1.06, 1.08)	—	1.04 (1.03, 1.04)	1.04 (1.03, 1.04)	1.04 (1.03, 1.04)	—
**Female**	0.69 (0.63, 0.76)	0.69 (0.63, 0.75)	0.69 (0.63, 0.75)	0.73 (0.67, 0.80)	0.70 (0.64, 0.77)	0.70 (0.64, 0.77)	0.70 (0.64, 0.78)	0.70 (0.64, 0.78)
**Race (ref: Non-Hispanic White)**								
Non-Hispanic Black	0.84 (0.74, 0.97)	0.85 (0.74, 0.97)	0.85 (0.74, 0.97)	0.86 (0.75, 0.99)	0.78 (0.67, 0.89)	0.78 (0.68, 0.90)	0.78 (0.67, 0.89)	0.76 (0.66, 0.88)
Hispanic	0.79 (0.66, 0.95)	0.79 (0.66, 0.95)	0.79 (0.66, 0.95)	0.82 (0.69, 0.97)	0.82 (0.68, 0.98)	0.82 (0.68, 0.98)	0.82 (0.68, 0.98)	0.84 (0.70, 1.01)
**Education**	1.00 (0.98, 1.01)	1.00 (0.98, 1.01)	1.00 (0.98, 1.01)	1.00 (0.98, 1.01)	0.99 (0.98, 1.01)	0.99 (0.98, 1.01)	0.99 (0.98, 1.01)	0.99 (0.98, 1.01)
**Married**	0.96 (0.87, 1.06)	0.96 (0.87, 1.06)	0.96 (0.87, 1.06)	1.12 (1.02, 1.23)	0.97 (0.88, 1.07)	0.97 (0.88, 1.07)	0.97 (0.88, 1.07)	0.91 (0.82, 1.00)
**Household wealth**								
2nd	0.96 (0.86, 1.07)	0.96 (0.86, 1.08)	0.96 (0.86, 1.08)	0.92 (0.83, 1.02)	1.01 (0.90, 1.14)	1.02 (0.90, 1.14)	1.02 (0.90, 1.14)	1.04 (0.92, 1.17)
3rd	0.84 (0.74, 0.95)	0.84 (0.74, 0.95)	0.84 (0.74, 0.95)	0.81 (0.72, 0.91)	0.91 (0.79, 1.03)	0.91 (0.80, 1.04)	0.91 (0.80, 1.04)	0.92 (0.80, 1.05)
**Medicaid**	1.04 (0.87, 1.24)	1.05 (0.88, 1.25)	1.05 (0.88, 1.25)	1.12 (0.94, 1.32)	1.04 (0.87, 1.24)	1.05 (0.88, 1.25)	1.04 (0.88, 1.24)	1.00 (0.84, 1.20)
**Dental visit**	0.80 (0.72, 0.88)	0.80 (0.72, 0.88)	0.80 (0.72, 0.88)	0.80 (0.72, 0.87)	0.81 (0.73, 0.89)	0.81 (0.73, 0.89)	0.81 (0.73, 0.89)	0.81 (0.73, 0.90)
**Exercise ≥ once/month**	0.61 (0.55, 0.67)	0.61 (0.55, 0.67)	0.61 (0.55, 0.67)	0.70 (0.63, 0.76)	0.74 (0.67, 0.82)	0.74 (0.66, 0.82)	0.73 (0.66, 0.81)	0.72 (0.65, 0.80)
**Smoking (ever)**	1.43 (1.31, 1.56)	1.43 (1.31, 1.56)	1.43 (1.31, 1.56)	1.50 (1.38, 1.63)	1.34 (1.23, 1.47)	1.34 (1.22, 1.47)	1.34 (1.23, 1.47)	1.29 (1.18, 1.42)
**Drinking (ever)**	0.92 (0.85, 1.01)	0.92 (0.85, 1.01)	0.92 (0.85, 1.01)	0.92 (0.85, 1.00)	0.94 (0.86, 1.03)	0.95 (0.86, 1.04)	0.94 (0.86, 1.03)	0.96 (0.87, 1.05)
**BMI (ref: normal weight)**								
Underweight	1.89 (1.36, 2.61)	1.88 (1.36, 2.61)	1.88 (1.36, 2.62)	1.88 (1.43, 2.48)	1.84 (1.31, 2.59)	1.84 (1.31, 2.59)	1.85 (1.31, 2.61)	1.79 (1.28, 2.49)
Overweight	0.83 (0.75, 0.92)	0.83 (0.75, 0.92)	0.83 (0.75, 0.92)	0.92 (0.83, 1.01)	0.89 (0.80, 0.99)	0.89 (0.80, 0.99)	0.89 (0.80, 0.99)	0.87 (0.79, 0.97)
Obese	0.94 (0.85, 1.05)	0.94 (0.85, 1.05)	0.94 (0.85, 1.05)	1.10 (0.99, 1.22)	1.02 (0.91, 1.14)	1.02 (0.91, 1.14)	1.02 (0.91, 1.14)	0.97 (0.87, 1.09)
**Heart disease**	1.46 (1.34, 1.60)	1.46 (1.34, 1.59)	1.46 (1.34, 1.60)	1.33 (1.23, 1.45)	1.34 (1.22, 1.47)	1.34 (1.22, 1.46)	1.34 (1.22, 1.47)	1.31 (1.20, 1.44)
**Stroke**	1.36 (1.19, 1.56)	1.36 (1.19, 1.56)	1.36 (1.19, 1.56)	1.23 (1.08, 1.39)	1.06 (0.91, 1.22)	1.06 (0.91, 1.22)	1.05 (0.91, 1.22)	1.05 (0.91, 1.21)
**Diabetes**	1.41 (1.27, 1.55)	1.40 (1.27, 1.55)	1.40 (1.27, 1.54)	1.44 (1.31, 1.58)	1.33 (1.20, 1.47)	1.33 (1.20, 1.47)	1.33 (1.20, 1.47)	1.28 (1.16, 1.42)

Abbreviations: BMI, body mass index; PRS, polygenic risk score.

a Competing risk: Incident dementia.

In contrast, the Fine–Gray models, which treated all-cause dementia as a competing risk, revealed a significant interaction between edentulism and PRS. While the main effect of edentulism was significant in Models 1 and 2, the final interaction Model 3 identified a significant synergistic interaction between edentulism and high PRS (sHR = 1.36; 95% CI, 1.00-1.84). This finding suggests that the risk or mortality was significantly amplified when both edentulism and a high genetic risk for dementia were present, an effect that became apparent only after accounting for dementia as a competing event. The cumulative incidence curves for mortality are displayed in Figure 2. Panel B presents Kaplan–Meier survival estimates for all-cause mortality, and Panel D displays cumulative incidence functions from the Fine–Gray competing risk model treating dementia as a competing event. Together, the curves depict differences across the four joint exposure groups defined by dentate status and PRS category, highlighting that individuals who were both edentulous and had a high PRS experienced the steepest increase in mortality risk over time.

### Sensitivity analyses

Sensitivity analyses that used age as the underlying time scale (Model 4) confirmed the robustness of our primary findings. For the dementia outcome, the significant attenuating interaction between edentulism and high PRS persisted in both Cox (HR = 0.74; 95% CI, 0.56-0.98) and Fine–Gray models (sHR = 0.70; 95% CI, 0.52-0.94). For the mortality outcome, the synergistic interaction in the Fine–Gray model remained significant and strengthened (sHR = 1.40; 95% CI, 1.03-1.90), underscoring the stability of our results.

## Discussion

In this nationally representative cohort of older U.S. adults followed for up to 17 years, we found that the relationship between edentulism, genetic susceptibility, and aging outcomes was more complex than a simple additive model of risk: the joint effects of edentulism and genetic susceptibility differed depending on the outcome examined. Specifically, when examining the four joint exposure groups defined by dentate status and PRS category, the increased dementia risk associated with high PRS was primarily observed among dentate individuals, whereas the risk of mortality was highest among those who were both edentulous and genetically susceptible.

Our stepwise modeling strategy, beginning with edentulism alone, then adding PRS, and finally introducing their interaction, revealed distinct layers of association. Edentulism alone was not significantly associated with incident dementia after adjustment for sociodemographic and health factors, suggesting that its cognitive effects are likely mediated through broader health pathways rather than direct neurodegeneration.^33–36^ The finding contrasts with previous research where edentulism was significantly associated with dementia risk.^37–39^ A plausible explanation could be that the inclusion of genetic risk may have explained variance that was previously attributed to edentulism, highlighting a more complex gene–environment interplay. When PRS was added, a modest but significant genetic effect on dementia risk emerged, confirming the role of polygenic susceptibility in late-life cognitive decline.^40^ However, once the interaction between edentulism and PRS was introduced, the narrative shifted: the detrimental effect of high PRS was most evident among dentate individuals when comparing the joint exposure groups, while it was markedly attenuated among those who were edentulous. In contrast, for mortality, the combination of edentulism and high PRS produced a synergistic effect, conferring the highest hazard of death. This sequence of findings demonstrates that genetic risk does not act in isolation but is shaped by an individual’s cumulative health burden and survival dynamics.^41^^,^^42^

The antagonistic interaction between edentulism and high PRS for dementia suggests that individuals with severe oral health deterioration, like edentulism, may experience a different aging trajectory, one characterized by higher early mortality that precludes dementia onset. This interpretation aligns with the competing risk framework: those at highest genetic risk for dementia may die early when compounded by edentulism-related systemic stress that accelerates aging and mortality, reducing the likelihood of surviving long enough to manifest dementia. Chronic oral inflammation, impaired nutrition, and immune dysregulation have been proposed as potential pathways linking tooth loss with systemic decline, while lifelong disadvantage reflected in edentulism may contribute to elevated allostatic load.^23^^,^^37^^,^^43^^,^^44^ These oral–systemic pathways may contribute to heightened mortality risk, which could partially explain the attenuated genetic association with dementia observed among edentulous individuals.^45^ The competing risk models, which explicitly account for mortality, confirmed this attenuation pattern, supporting the notion of mortality selection rather than neuroprotection. Importantly, this pattern should not be interpreted as evidence that edentulism biologically reduces genetic susceptibility to dementia. Rather, it likely reflects the differential timing of events under competing risks. Individuals with both high genetic susceptibility and edentulism may experience elevated mortality risk and, therefore, may be less likely to survive long enough to receive a dementia diagnosis. In this sense, the observed interaction is consistent with survival dynamics rather than a biological suppression of genetic vulnerability.

Alternatively, edentulism may serve as a proxy for multimorbidity and cumulative physiological decline that overshadows the contribution of genetic risk to neurodegeneration. It reflects the endpoint of a lifetime of dental disease and the individual’s absence of adequate dental treatment, particularly preventive or restorative care.^46^ Chronic inflammation, malnutrition, and vascular pathology associated with tooth loss have been suggested as contributors to frailty and mortality, potentially narrowing the time window during which dementia can clinically manifest.^23^^,^^43^ Additionally, a ceiling effect may explain the non-significant association between edentulism and dementia. At advanced ages, dementia incidence is already high regardless of health conditions. Among the oldest-old, the likelihood of developing dementia is substantial, limiting the additional explanatory power of edentulism.^10^^,^^47^ These findings reinforce the need to view edentulism as part of a broader constellation of aging processes, in which biological, behavioral, and social vulnerabilities interact dynamically over time to shape health trajectories.

Our results further underscore that edentulism is not merely an oral health condition but a biological embodiment of cumulative disadvantage. In this context, edentulism in the present study should be interpreted less as a direct biological exposure and more as a proxy indicator of accumulated life-course disadvantage and systemic health burden. Consistent with prior literature, edentulous participants in our study were older, poorer, and more likely to have cardiometabolic comorbidities and limited healthcare access.^7^^,^^48^^,^^49^ Such clustering of disadvantage likely reflects lifelong exposure to resource constraints, stress, and inflammation, all of which are linked to accelerated biological aging. Importantly, these systemic burdens can shorten survival independent of genetic dementia risk, explaining why mortality risk is amplified even as dementia incidence appears attenuated among edentulous individuals.

In this context, the observation that high PRS exerts a weaker association with dementia among edentulous individuals does not imply protection. Rather, it reflects a stage of physiological decline where the predictive value of genetic information diminishes because death intervenes first. Conversely, among relatively healthier, dentate older adults, a dementia PRS may have its greatest clinical and preventive utility. In this group, who lack overt markers of systemic decline, a high PRS identifies individuals at substantially elevated risk for dementia who might otherwise appear low-risk based on clinical presentation alone. These findings may have implications for emerging precision prevention approaches. Genetic risk information may be informative in disease prevention among relatively healthier older adults who do not exhibit overt markers of systemic decline. Please note that the present results are relative risk patterns rather than absolute risk differences required for clinical risk stratification. Taken together, these findings suggest that the translation of genetic information into meaningful clinical or public health action must consider the biological and social context of aging.

Beyond their substantive implications, these findings demonstrate the necessity of a competing risk framework in gene–environment studies of aging. By modeling dementia and death simultaneously, we reveal interaction patterns that would remain obscured in standard survival analyses. The combination of high genetic susceptibility and poor oral systemic health proved particularly detrimental, suggesting potential shared biological mechanisms, such as chronic inflammation, immune dysregulation, and vascular compromise, that contribute to both neurodegeneration and mortality.^37^^,^^44^ Identifying biomarkers along these intersecting pathways represents a promising direction for future research.

This study benefits from a large population-based cohort with linked genetic and Medicare claims data, enabling precise identification of dementia onset. The use of both Cox and Fine–Gray models provides complementary perspectives on cause-specific hazards and cumulative risk in the presence of competing mortality. Nevertheless, several limitations merit consideration. Edentulism was self-reported and assessed intermittently, although logical imputation likely minimized misclassification.^28^^,^^50^ The HRS measures dental care utilization only as a binary indicator of whether a dental visit occurred, without information on the specific reason for the visit (e.g., preventive care, denture maintenance, or treatment). The absence of such granular data prevented us from examining how specific clinical dimensions, such as functional chewing ability or prosthetic success, influence diverging aging outcomes. Consequently, while edentulism serves as a powerful proxy for cumulative disadvantage, future studies with more detailed oral health measures are needed to fully disentangle how functional status shapes the balance between dementia risk and survival. Second, the PRS, while state of the art, may still capture genetic risk incompletely, particularly for non-European ancestry groups.^40^ Although the HRS constructed ancestry-specific PRS using IGAP-derived weights, differences in linkage disequilibrium structure, allele frequencies, and representation in genome-wide association studies may influence predictive performance across populations. Thus, the interpretation of gene–environment interactions involving PRS should be considered with caution in diverse populations. Third, residual confounding by unmeasured socioeconomic or health factors, such as early-life socioeconomic adversity, dietary quality, or subclinical inflammatory burden, cannot be fully excluded. Another consideration is the potential for temporal ambiguity between tooth loss and cognitive decline. Although participants with prevalent dementia at baseline were excluded and a washout period was applied to identify incident dementia, tooth loss in late life may partly reflect prodromal cognitive or functional decline that reduces self-care behaviors or dental service utilization. As a result, the relationship between edentulism and cognitive outcomes may be bidirectional. This possibility could contribute to the observed associations with dementia risk. However, the stronger interaction observed for mortality supports the interpretation that survival dynamics play an important role. Individuals with both edentulism and high genetic susceptibility may experience elevated mortality, which reduces the probability of surviving long enough for dementia to be clinically diagnosed. Future studies with earlier-life oral health measures and longitudinal tracking of dental status could further clarify the temporal ordering of these relationships.

## Conclusions

Edentulism and polygenic risk for dementia jointly shape aging trajectories in outcome-specific and conceptually divergent ways. Rather than amplifying genetic vulnerability to dementia, edentulism appears to shift the balance between dementia incidence and survival—attenuating observed dementia risk but amplifying mortality among genetically susceptible individuals. These findings emphasize that genetic risk operates within a broader ecosystem of health, frailty, and social context. Future studies should focus on the shared biological pathways linking oral health, systemic inflammation, and neurodegeneration, with the goal of developing integrated prevention strategies that bridge precise medicine and population health.

## Supplementary material

Supplementary material is available at *Innovation in Aging* online.

## Supplementary Material

igag052_Supplementary_Data

## Data Availability

This study uses secondary data analysis of existing datasets and was not preregistered. The data used in this study are only available to approved users.
