# Supplementary material for: Divergent effects of oral health and genetic risk: competing risks of dementia and mortality
Source: Innov Aging. 2026 May 7;10(7):igag052. doi: 10.1093/geroni/igag052 (PMC13274639; doi:10.1093/geroni/igag052)
Supplement: igag052_Supplementary_Data [file igag052_supplementary_data.pdf]

Innovation in Aging **Supplementary Material: Liu, Qi, Liu, Luo, Xu, & Wu. Divergent effects of oral health and genetic risk: competing risks of dementia and mortality.**

Supplementary Table 1. ICD-9 and ICD-10 Diagnostic Codes Used to Identify Incident Dementia.

**Diagnostic Codes**				
**ICD-9**			**ICD-10**	
290	Dementias		A81.0	Sporadic Creutzfeldt-Jakob disease (UKB)
290	Senile dementia, uncomplicated		F00	Dementia in Alzheimer's disease
290.1	Presenile dementia		F00.0	Dementia in Alzheimer's disease with early onset
290.1	Presenile dementia, uncomplicated		F00.1	Dementia in Alzheimer's disease with late onset
290.11	Presenile dementia with delirium		F00.2	Dementia in Alzheimer's disease, atypical or mixed type
290.12	Presenile dementia with delusional features		F00.9	Dementia in Alzheimer's disease, unspecified
290.13	Presenile dementia with depressive features		F01	Vascular dementia
290.2	Senile dementia with delusional or depressive features		F01.0	Vascular dementia of acute onset
290.2	Senile dementia with delusional features		F01.1	Multi-infarct dementia
290.21	Senile dementia with depressive features		F01.2	Subcortical vascular dementia
290.3	Senile dementia with delirium		F01.3	Mixed cortical and sub-cortical vascular dementia
290.4	Vascular dementia		F01.5	Vascular dementia
290.4	Vascular dementia, uncomplicated		F01.50	Vascular dementia without behavioral disturbance
290.41	Vascular dementia with delirium		F01.51	Vascular dementia with behavioral disturbance
290.42	Vascular dementia with delusions		F01.8	Other vascular dementia
290.43	Vascular dementia with depressed mood		F01.9	Vascular dementia, unspecified
290.8	Other specified senile psychotic conditions		F02	Dementia in other diseases classified elsewhere
290.9	Unspecified senile psychotic condition		F02.0	Dementia in Picks disease
291.1	Alcohol-induced persisting amnestic disorder		F02.1	Dementia in Creutzfeldt-Jakob disease
291.2	Alcohol-induced persisting dementia		F02.2	Dementia in Huntington's disease
292.82	Drug-induced persisting dementia		F02.3	Dementia in Parkinsons' disease
292.83	Drug-induced persisting amnestic disorder		F02.4	Dementia in HIV disease
293	Delirium due to conditions classified elsewhere		F02.8	Dementia in other diseases classified elsewhere
293.1	Subacute delirium		F02.80	Dementia in other diseases classified elsewhere without behavioral disturbance
293.9	Unspecified transient mental disorders due to conditions classified elsewhere, other		F02.81	Dementia in other diseases classified elsewhere with behavioral disturbance
294	Amnestic disorder classified elsewhere (Korsakoff)		F03	Unspecified dementia
294.1	Dementia in conditions classified elsewhere		F03.9	Unspecified dementia
294.1	Dementia in conditions classified elsewhere without behavioral disturbance		F03.90	Unspecified dementia
294.11	Dementia in conditions classified elsewhere with behavioral disturbance		F03.91	Unspecified dementia
294.2	Dementia, unspecified		F04	Amnestic disorder due to known physiological condition
294.2	Dementia, unspecified without behavioral disturbance		F05	Delirium due to known physiological condition
294.21	Dementia, unspecified with behavioral disturbance		F05.1	Delirium superimposed on dementia
294.8	Other persistent mental disorders due to conditions classified elsewhere		F06.1	Catatonic disorder due to known physiological condition
294.9	Unspecified persistent mental disorders due to conditions classified elsewhere		F06.8	Other specified mental disorders due to known physiological condition
331	Alzheimer's disease		F10.26	Alcohol dependence with alcohol induced persisting amnestic disorder
331.1	Pick's Disease		F10.27	Alcohol dependence with alcohol induced persisting dementia

Diagnostic Codes				
**ICD-9**			**ICD-10**	
331.11	Pick's Disease		F10.6	Mental and behavioral disorders due to use of alcohol-amnesic syndrome
331.19	Other frontotemporal dementia		G13.8	Systemic atrophy affecting central nervous systems in other diseases classified elsewhere
331.2	Senile degeneration of brain		G30	Alzheimer's disease
331.5	Idiopathic normal pressure hydrocephalus		G30.0	Alzheimer's disease with early onset
331.7	Cerebral degeneration in diseases classified elsewhere		G30.1	Alzheimer's disease with late onset
331.8	Other degenerative diseases of nervous system		G30.8	Alzheimer's disease, other
331.82	Dementia with Lewy bodies		G30.9	Alzheimer's disease, unspecified
331.89	Other cerebral degeneration		G31.0	Frontotemporal dementia
331.9	Cerebral degeneration, unspecified		G31.01	Pick's disease
797	Senility without mention of psychosis		G31.09	Other frontotemporal dementia
			G31.1	Senile degeneration of brain, not elsewhere classified
			G31.2	Degeneration of nervous system due to alcohol
			G31.8	Other specified degenerative diseases of nervous system
			G31.83	Dementia with Lewy bodies
			G31.89	Other specified degenerative diseases of nervous system
			G31.9	Degenerative disease of nervous system, unspecified
			G32	Other degenerative disorders of nervous system in diseases classified elsewhere
			G32.0	Subacute combined degeneration of spinal cord in diseases classified elsewhere
			G32.8	Other specified degenerative disorders of nervous system in diseases classified elsewhere
			G32.81	Cerebellar ataxia in diseases classified elsewhere
			G32.89	Other specified degenerative disorders of nervous system in diseases classified elsewhere
			G94	Other disorders of brain in diseases classified elsewhere
			I67.3	Binswanger's disease
			R41.81	Age-related cognitive decline
			R54	Age-related physical debility

Symptoms associated with dementia				
**ICD-9**			**ICD-10**	
780.93	Amnesia		R41.1	Anterograde amnesia
784.3	Aphasia		R41.2	Retrograde amnesia
784.69	Other symbolic dysfunctions, apraxia, agnosia		R41.3	Other amnesia
331.83	Mild cognitive impairment, so stated		R47.01	Aphasia
			R48.1	Agnosia
			R48.2	Apraxia
			R48.8	Other symbolic dysfunctions
			G31.84	Mild cognitive impairment, so stated

Supplementary Table 2. Comparison of Baseline Characteristics between the Analytical Cohort and Participants Excluded due to Prevalent Dementia.

**Variables**	**All** *N*=10,369	**Included** *n*=9,806 (94.57%)	**Excluded** *n*=563 (5.43%)	***p***
Edentulism	2,052 (19.79%)	1,901 (19.39%)	151 (26.82%)	<.001
Polygenic Risk Score				.328
Low	2,091 (20.17%)	1,972 (20.11%)	119 (21.14%)	
Intermediate	6,231 (60.09%)	5,909 (60.26%)	322 (57.19%)	
High	2,047 (19.74%)	1,925 (19.63%)	122 (21.67%)	
Age	72.49 (6.30)	72.21 (6.11)	77.36 (7.49)	<.001
Female	5,995 (57.82%)	5,666 (57.78%)	329 (58.44%)	.759
Race				.502
Non-Hispanic White	8.014 (77.29%)	7,584 (77.34%)	430 (76.38%)	
Non-Hispanic Black	1,423 (13.72%)	1,337 (13.63%)	86 (15.28%)	
Hispanic	932 (8.99%)	885 (9.03%)	47 (8.35%)	
Education	12.44 (3.20)	12.47 (3.17)	11.94 (3.67)	<.001
Married	6,700 (64.62%)	6,373 (65.00%)	327 (58.08%)	.001
Household wealth				<.001
1st	2,929 (28.25%)	2,707 (27.61%)	222 (39.43%)	
2nd	3,470 (33.47%)	3,304 (33.69%)	166 (29.48%)	
3rd	3,970 (38.29%)	3,795 (38.70%)	175 (31.08%)	
Medicaid	847 (8.22%)	774 (7.94%)	73 (13.13%)	<.001
Dental visit	6,525 (63.04%)	6,202 (63.36%)	323 (57.47%)	.005
Exercise ≥once/month	8,118 (78.39%)	7,783 (79.48%)	335 (59.50%)	<.001
Smoking (ever)	5,873 (57.05%)	5,551 (57.02%)	322 (57.60%)	.785
Drinking (ever)	5,297 (51.10%)	5,078 (51.80%)	219 (38.90%)	<.001
Body Mass Index				<.001
Normal weight	2,956 (28.51%)	2,745 (27.99%)	211 (37.48%)	
Underweight	133 (1.28%)	117 (1.19%)	16 (2.84%)	
Overweight	3,975 (38.34%)	3,771 (38.46%)	204 (36.23%)	
Obese	3,305 (31.87%)	3,173 (32.36%)	132 (23.45%)	
Heart disease	2,846 (27.45%)	2,614 (26.66%)	232 (41.21%)	<.001
Stroke	917 (8.84%)	790 (8.06%)	127 (22.56%)	<.001
Diabetes	2,365 (22.81%)	2,217 (22.61%)	148 (26.29%)	<.001

Note: Data are expressed as Mean (Standard Deviation [SD]) for continuous variables and N (%) for categorical variables. Percentages have been rounded and may not total 100, and numbers may not total numbers in column headings owing to missing data.

Comparisons were performed using a t-test for continuous variables and the χ^2 test for categorical variables.
